# Supplementary material for: A single-cell spatial chart of the airway wall reveals proinflammatory cellular ecosystems and their interactions in health and asthma
Source: Nat Immunol. 2025 May 21;26(6):920–33. doi: 10.1038/s41590-025-02161-3 (PMC12133579; doi:10.1038/s41590-025-02161-3)
Supplement: Supplementary file 1 — Reporting Summary [file 41590_2025_2161_MOESM1_ESM.pdf]

nature portfolio

Corresponding author(s): Dr. Régis Joulia

Last updated by author(s): Mar 31, 2025

Reporting Summary

Nature Portfolio wishes to improve the reproducibility of the work that we publish. This form provides structure for consistency and transparency in reporting. For further information on Nature Portfolio policies, see our Editorial Policies and the Editorial Policy Checklist.

Statistics

For all statistical analyses, confirm that the following items are present in the figure legend, table legend, main text, or Methods section.

n/a	Confirmed	
☐	☒	The exact sample size (*n*) for each experimental group/condition, given as a discrete number and unit of measurement
☐	☒	A statement on whether measurements were taken from distinct samples or whether the same sample was measured repeatedly
☐	☒	The statistical test(s) used AND whether they are one- or two-sided<br>*Only common tests should be described solely by name; describe more complex techniques in the Methods section.*
☒	☐	A description of all covariates tested
☐	☒	A description of any assumptions or corrections, such as tests of normality and adjustment for multiple comparisons
☐	☒	A full description of the statistical parameters including central tendency (e.g. means) or other basic estimates (e.g. regression coefficient) AND variation (e.g. standard deviation) or associated estimates of uncertainty (e.g. confidence intervals)
☐	☒	For null hypothesis testing, the test statistic (e.g. *F*, *t*, *r*) with confidence intervals, effect sizes, degrees of freedom and *P* value noted<br>*Give P values as exact values whenever suitable.*
☒	☐	For Bayesian analysis, information on the choice of priors and Markov chain Monte Carlo settings
☒	☐	For hierarchical and complex designs, identification of the appropriate level for tests and full reporting of outcomes
☒	☐	Estimates of effect sizes (e.g. Cohen's *d*, Pearson's *r*), indicating how they were calculated

Our web collection on statistics for biologists contains articles on many of the points above.

Software and code

Policy information about availability of computer code

Data collection	LEICA LAS (version X), Xenium platform (v3.0), Nanostring DSP (V3.0.0.111)
Data analysis	Imaris (v10.1), LEICA LAS version X, R studio (version 2023.06.01), Xenium explorer (version 3.1.1, 10X), Prism software (GraphPad v9.4.1), R (v4.3.0), Python(v3.12), Nanostring DSP analysis suite (GEOMX-B0007), UMAP (0.2.10.0), ggplot2 (3.5.1), sctransform (0.4.1), Seurat (5.2.1), phenoptr (0.3.2), drug2cell (v0.1.0), SquidPy (v1.4.1), GSVA (2.0.1), Stringr (1.5.1), Dplyr (1.1.4)

For manuscripts utilizing custom algorithms or software that are central to the research but not yet described in published literature, software must be made available to editors and reviewers. We strongly encourage code deposition in a community repository (e.g. GitHub). See the Nature Portfolio guidelines for submitting code & software for further information.

Data

Policy information about availability of data

All manuscripts must include a data availability statement. This statement should provide the following information, where applicable:

- Accession codes, unique identifiers, or web links for publicly available datasets
- A description of any restrictions on data availability
- For clinical datasets or third party data, please ensure that the statement adheres to our policy

The analysis was performed using published and freely available software and code mentioned in relevant method sections. Raw and processed data are available at

on the NIH GEO online database under GSE269354. The ChEMBL database used for Drug2cell spatial analysis is available at https://ftp.ebi.ac.uk/pub/databases/chembl/ChEMBLdb/latest/ . Single cell RNA-seq data are available on the Sanger Institute database (https://5locationslung.cellgeni.sanger.ac.uk/all). Nanostring raw data and Q3 norm data are available as Source data. Further information and requests for resources and reagents should be directed to and will be fulfilled by lead author Régis Joulia (r.joulia@imperial.ac.uk).

Research involving human participants, their data, or biological material

Policy information about studies with human participants or human data. See also policy information about sex, gender (identity/presentation), and sexual orientation and race, ethnicity and racism.

Reporting on sex and gender	We analysed samples from 17 females and 9 males, this is reflective of the availability of donors and the predispostion of asthma in women. KIA samples information are available from the oringinal publication of the clinical trial (ClinicalTrials.gov number, NCT01097694).
Reporting on race, ethnicity, or other socially relevant groupings	Tissues from the first cohort are from the UK population, mainly urban location around London and Leicester. The samples from the KIA study were colelcted in North america and ethnics information can be found in the original publication and clinical trial.
Population characteristics	Adult patients (18-65 years old) with severe asthma as defined by European Respiratory Society (ERS) and American Thoracic Society (ATS) guidelines were recruited. All had a BMI of 18.5-35 kg/m2 expect one at 66.11, non-smoker or ex-smoker for at least past 12 months, FEV1 of ≥1 litre AND ≥ 60% predicted. For patient stratification between mild and severe asthma, we employed the European Respiratory Society (ERS) and American Thoracic Society (ATS) guidelines. Mild asthma was characterised by infrequent symptoms that can be managed with low-dose medications. In contrast, moderate to severe asthma is defined by persistent, heavy symptoms despite high-dose medications, frequent exacerbations, and often requires additional therapies such as biologics.
Recruitment	All lung tissues were obtained after Research Ethics Committee approvals and informed consent from the donor families.
Ethics oversight	The collection of endobronchial biopsies was approved by London - Bloomsbury Research Ethics Committee under the approval REC 19/LO/1675.<br>KIA clinical trial was approved by the Institutional Review Board (IRB) or Independent Ethics Committee (IEC) of Harvard MEdical School<br>Human adult samples used in this research project were obtained from the Imperial College Healthcare Tissue Bank (ICHTB). ICHTB is supported by the National Institute for Health Research (NIHR) Biomedical Research Centre based at Imperial College Healthcare NHS Trust and Imperial College London. ICHTB is approved by Wales REC3 to release human material for research (17/WA/0161), and the samples for this project (R22006) were issued from sub-collection reference number ICB_NC_21_017. The views expressed are those of the authors and not necessarily those of the NHS, the NIHR or the Department of Health. The biopsy samples from University Hospitals of Leicester were issued under MTA 2021S-0809-2029 between University of Leicester/UHL and Imperial College London and were approved by the research ethics committee MREC: 08/H0406 and IRAS: 8824.

Note that full information on the approval of the study protocol must also be provided in the manuscript.

Field-specific reporting

Please select the one below that is the best fit for your research. If you are not sure, read the appropriate sections before making your selection.

☒ Life sciences ☐ Behavioural & social sciences ☐ Ecological, evolutionary & environmental sciences

For a reference copy of the document with all sections, see nature.com/documents/nr-reporting-summary-flat.pdf

Life sciences study design

All studies must disclose on these points even when the disclosure is negative.

Sample size	Sample size was determined to provide meaningfull data with this new technology. As nobody reported this type of data before, we estimated that 4 patients per group will give enough power and this aligned with Human Cell Atlas crtieria.
Data exclusions	No data were excluded
Replication	We replicated our spatial single cell RNAseq experiment in 3 independant cohorts and different technologies with similar results. Human imaging data such as PCLS were performed on separate part of the tissue. Mutiple fields of view (at least 5) were captured for each part of the tissue. All attempts at replication were successful.
Randomization	To avoid batch effects, we run simulatenously samples from healthy and asthma donors or palcebo and imatinib samples. Original clinicial trial includes randomization of patients receiving placebo or imatinib.
Blinding	Investigators were blinded during data collection for Xenium and GeoMX datasets. No blinding was required for imaging lung samples as no separate groups were compared. Samples were analysed as a whole before being analysed considering the pathology status as such reducing any potential biased. Furthermore, we applied multiple unbaised analysis strategy to discover new elements of lung biology.

Reporting for specific materials, systems and methods

We require information from authors about some types of materials, experimental systems and methods used in many studies. Here, indicate whether each material, system or method listed is relevant to your study. If you are not sure if a list item applies to your research, read the appropriate section before selecting a response.

Materials & experimental systems

n/a	Involved in the study
☐	☒ Antibodies
☒	☐ Eukaryotic cell lines
☒	☐ Palaeontology and archaeology
☒	☐ Animals and other organisms
☒	☐ Clinical data
☒	☐ Dual use research of concern
☒	☐ Plants

Methods

n/a	Involved in the study
☒	☐ ChIP-seq
☒	☐ Flow cytometry
☒	☐ MRI-based neuroimaging

Antibodies

Antibodies used

Alexa Fluor 488 anti-human CD31 (clone WM59, dilution 5 ug/ml) from Biolegend, avidin (#A887) was conjugated to Alexa fluor 647 (#A20186) dilution 3.3 ug/ml, ThermoFisher.

Validation

Validation of all primary commercial antibodies for the species and application was warranted by the vendors. Validation statement can be found on the manufacturers' website. For Alexa Fluor 488 anti-human CD31, each lot of this antibody is quality control tested by immunofluorescent staining with flow cytometric analysis. Clone WM59 has been reported to recognize the D2 extracellular portion of CD31. For avidin home made conjugate, in house validation was performed using primary mast cell expressing strongly the recognsied antigen (i.e. heparin).

Plants

Seed stocks

Report on the source of all seed stocks or other plant material used. If applicable, state the seed stock centre and catalogue number. If plant specimens were collected from the field, describe the collection location, date and sampling procedures.

Novel plant genotypes

Describe the methods by which all novel plant genotypes were produced. This includes those generated by transgenic approaches, gene editing, chemical/radiation-based mutagenesis and hybridization. For transgenic lines, describe the transformation method, the number of independent lines analyzed and the generation upon which experiments were performed. For gene-edited lines, describe the editor used, the endogenous sequence targeted for editing, the targeting guide RNA sequence (if applicable) and how the editor was applied.

Authentication

Describe any authentication procedures for each seed stock used or novel genotype generated. Describe any experiments used to assess the effect of a mutation and, where applicable, how potential secondary effects (e.g. second site T-DNA insertions, mosiacism, off-target gene editing) were examined.
